# Effects of Vitamin D Levels on Long-Term Coronary Events in Patients with Proven Coronary Artery Disease: Six-Year Follow-Up

**DOI:** 10.3390/jcm12216835

**Published:** 2023-10-29

**Authors:** Aysun Erdem Yaman, Ufuk Sadık Ceylan

**Affiliations:** Siyami Ersek Thoracic Surgery Research and Training Hospital, Department of Cardiology, 34668 Istanbul, Turkey

**Keywords:** vitamin D, coronary artery disease, mortality, myocardial infarction

## Abstract

Although some clinical studies have claimed that low-dose vitamin D (Vit-D) increases the risk of long-term cardiac events, in others, no association was found. To better understand the impact of Vit-D levels on long-term cardiac events in coronary artery disease patients, this study was designed. There were 408 patients with coronary artery disease (CAD). The patients were separated into three groups based on their Vit-D levels: group 1 had levels below 10 ng/mL, group 2 had levels between 10 and 20, and group 3 had levels above 20 ng/mL. Six years were spent monitoring the patients for non-fatal MI, death, vascular revascularization, and stable course data. Mortality was found to be similar between groups (group 1: 24.5%; group 2: 13.8%; group 3: 17.4%; *p* > 0.05). In group 3, 47.8% of the patients did not experience any cardiac event, while 28.7% in group 2 and 27.6% in group 1 did not experience any cardiac event, and these values were found to be significant in favor of group 3 (*p* = 0.006). Group 3 was found to have considerably lower rates of non-ST-elevated myocardial infarction (non-STEMI) and unstable angina (UA) than the other groups did (group 1: 49%; group 2: 38%; group 3: 27%; *p* = 0.001). In conclusion, although vitamin D deficiency does not accompany an increase in mortality, it is associated with an increase in non-STEMI and UA in patients who have previously been diagnosed with CAD.

## 1. Introduction

Coronary atherosclerosis is initiated by endothelial damage caused by risk factors, such as diabetes, hypertension, smoking, hyperlipidemia, and obesity. Experimental studies have demonstrated the crucial role of inflammation in the pathophysiological complexity of atherosclerotic disease. When the pathophysiology of the disease is examined, the first physiological change that emerges is the disruption of the endothelium [1]. NF-KB becomes active in the endothelium exposed to oxidized LDL. This causes the endothelium to upregulate proteins such as tissue factor (TF). Thus, the protective phenotype of the endothelium changes [2]. Endothelial dysfunction activates the renin–angiotensin system. Angiotensin II is produced in excess, and less nitric oxide is produced owing to endothelial dysfunction, which is linked to cardiovascular risk factors. Angiotensin II has an impact on endothelial function, inflammation, fibrinolytic balance, and plaque stability, which all affect atherosclerotic plaque progression [1]. Complication (ulceration, fissure or rupture) of an advanced atherosclerotic lesion causes exposure of intra-plaque TF. At the same time, circulating oxidized LDLs cause surface expression of TF and adhesion molecules in endothelial cells. While inflammation increases on the one hand, on the other hand, TF triggers coagulation factors in the circulation and intra-coronary thrombosis develops [2].

Vitamin D deficiency has increased in the general population. The role of Vit-D in calcium and bone metabolism is well established. Interestingly, vitamin D has also been shown to directly or indirectly regulate the cytokine levels, inflammation, renin–angiotensin system, and adaptive immune responses of inflammation. In many studies, 25 (OH) D3 levels lower than 20 ng/mL define vitamin D deficiency, 20–30 ng/mL define insufficiency, and values higher than 30 ng/mL are defined as normal [3].

Vit-D has anti-inflammatory properties, inhibits the macrophage clearance of cholesterol, and prevents the production of foam cells on vessel walls. It increases the level of nitric oxide by increasing the activity of endothelial nitric oxide synthase (eNOS), which facilitates nitric oxide production. The eNOS activity is associated with endothelial cell permeation, vascular tone change, and pro-thrombotic and pro-coagulation activities. Although vitamin D reduces the number of pro-inflammatory type 1 cytokines (interleukin 12(IL-12), IL-6, IL-8, Tumor necrosis factor alfa (TNF-α)), it increases the number of anti-inflammatory type 2 cytokines (IL-4, IL-5, IL-10) [4,5]. Thus, Vitamin D regulates the activity of NF-ΚB and STAT3, which are intracellular signals, and ensures that the endothelial cell remains in a protective phenotype [2,6]. In addition to the suppression of proatherogenic T lymphocytes and inhibition of smooth muscle cell production, Vitamin D has a vascular protection effect against advanced glycation products.

Possible mechanisms by which vitamin D deficiency may contribute to cardiovascular disease include increased renin–angiotensin levels, impaired insulin sensitivity, and increased inflammation. A strong link has been found between vitamin D deficiency and subclinical atherosclerosis and endothelial dysfunction in patients with near normal coronary arteries undergoing coronary angiography. Low Vit-D levels have promoted atherosclerosis, leading to vasculitis, foam cell formation, endothelial dysfunction, and smooth muscle cell proliferation [7,8]. Studies have shown that Vit-D has a strong endocrine inhibitory effect on renin synthesis. It has been observed that increased renin increases the incidence of atherosclerosis in the aortic arch, thoracic aorta, and abdominal aorta by 2–8 times, and macrophage infiltration increases in these plaques. There is evidence that Vit-D deficiency can increase parathyroid serum levels, and it may also have a direct stimulatory effect on renin secretion.

In various studies, Vit-D deficiency has been related to increased risks of ischemic heart disease, sudden death, myocardial infarction, and fatal ischemic heart disease/fatal myocardial infarction. The incidence of cardiovascular events during the five-year follow-up period was found to be 1.62 times higher in individuals with a mean age of 59 years who had vitamin D levels below 15 ng/mL after adjustment for conventional risk factors. Similarly, a strong relationship between basal vitamin D levels and cardiovascular mortality was observed in older adults (>65 years) during a seven-year follow-up [8,9]. In young patients who have survived after ST-elevation myocardial infarction, a reduction in vitamin D binding protein levels have been linked to an escalation in the number of coronary arteries that were affected [7]. In acute coronary syndrome patients, lower Vit-D levels (<7.3 ng/mL) are associated with severe long-term cardiovascular events. It has been reported that this relationship is especially seen in patients who are frequently hospitalized owing to acute coronary syndrome or decompensated heart failure. Additionally, the lowest-quartile vitamin D level was found to be a strong predictor of one-year mortality in patients with acute myocardial infarction [10].

In another study, it was shown that there was no relationship between the degree of stenosis in coronary artery disease and vitamin D deficiency [11]. A prospective study conducted on postmenopausal women with vitamin D deficiency who received Vit-D for four months found no change in their endothelial function or arterial stiffness, and Vit-D supplementation also failed to treat cardiovascular disease. Furthermore, the Women’s Health Study also found no relationship between levels of vitamin D deficiency and rates of myocardial infarction or angina [9].

Vit-D’s effect on cardiovascular events in CAD is controversial in the literature. Additionally, studies on the effect of vitamin D on long-term cardiac events in outpatient clinic patients who have previously undergone coronary intervention (PTCA or coronary bypass) are limited. Therefore, our aim was to investigate the association between vitamin D levels and cardiovascular events in patients diagnosed with CAD and followed up in an outpatient clinic.

## 2. Materials and Methods

### 2.1. The Study Protocol

**Figure 1:**
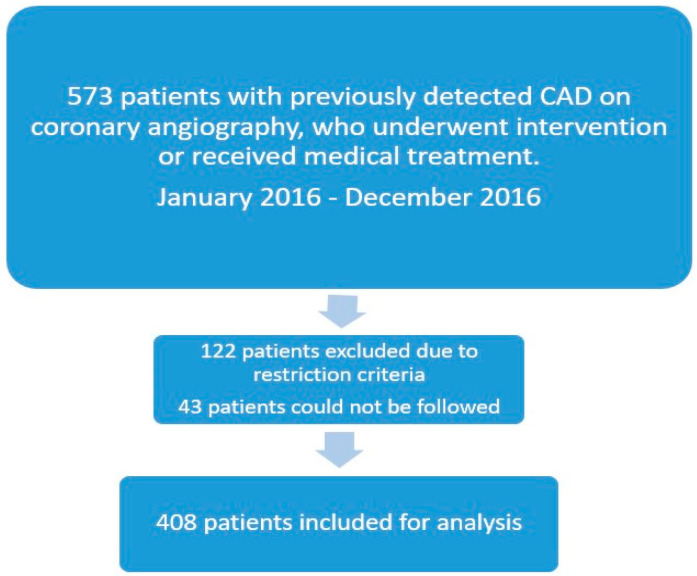
Study flowchart.

This observational retrospective study was conducted at Dr. Siyami Ersek Thoracic and Cardiovascular Surgery Training and Research Hospital, Turkey. A total of 4617 outpatient clinic patients were examined between January 2016 and December 2016. A study flowchart is depicted in Figure 1. A total of 573 individuals were chosen in a sequential manner from those who had undergone coronary artery bypass grafting, had undergone PTCA and stenting, or had been previously diagnosed with coronary heart disease and simply received medical care. On the basis of the study’s exclusion criteria, 122 patients were not included. Forty-three patients could not be followed up on; thus, they were omitted from the study. According to their Vit-D levels, 408 patients whose annual vitamin D measurements remained in the same range during the follow-up were split into three groups: group 1 had a Vit-D level below 10 ng/mL, group 2 had one between 10 and 20, and group 3 had one above 20 ng/mL. Clinical information was gathered from outpatient and hospital admissions electronic records. Phone calls were made to all the patients.

The patients included in the study had previously undergone PTCA and stenting, coronary artery bypass surgery in our hospital, or were followed up with medical treatment after coronary angiography previously performed in our hospital. The patient population is composed of patients living in the northwestern region of our country, and patients from foreign countries or with ethnic differences were not included in our patient group. Their BMI, whether they had a sedentary lifestyle, Mediterranean diet habits, smoking, alcohol use, disease, and medication use data were taken from the patients’ electronic records. The CDC’s definition of heavy alcohol consumption was used, and these patients were excluded from the study [12]. Fifteen patients who consumed excessive alcohol were identified. Patients with a family history of sudden cardiac death or a family history of premature coronary artery disease were not included in our study as this may have affected our study results. There were 23 of these patients, and they were not included in our study.

Liver illness; hyperparathyroidism; the usage of medications, like calcium and Vit-D; a history of malignancy; inflammatory bowel disease; a family history of sudden cardiac death; a family history of premature coronary artery disease; patients experiencing multiple coronary events of different types; and excessive alcohol consumption were among the exclusion criteria.

The clinical events were classified as mortality, urgent revascularization due to myocardial infarction, elective coronary revascularization resulting from non-STEACS, UA, chronic causes, and stable state, which means that there were no cardiac complaints. The definitions of ACS, ST-elevated myocardial infarction (STEMI), non-STEACS, and UA were used in accordance with the European Society of Cardiology guidelines [13].

The American Diabetes Association’s definition of diabetes mellitus was used to determine whether a subject had the condition [14]. A subject was defined as being affected by hypertension if the patient had a previous diagnosis, an arterial pressure >140/90 mm Hg, or underwent chronic treatment with anti-hypertensive medication [15]. A sedentary lifestyle is defined as lying down or sitting for more than 6 h a day on average [16]. All the patients were followed and treated in accordance with the guidelines in terms of additional diseases [17,18].

The current study was conducted in accordance with the Declaration of Helsinki and applicable local regulations. The local ethics committee gave the authors permission to proceed (approval number: 2023/50-4134). All the individuals provided informed consent before enrollment.

### 2.2. Laboratory Measurements

A blood sample was taken during the first hospital admission (Coulter LH780, Beckman Coulter Ireland Inc., Mervue, Galway, Ireland). Patients’ medication information was obtained from their electronic prescriptions.

### 2.3. Statistical Analysis

NCSS (Number Cruncher Statistics System) statistics software, from NCSS LLC, Kaysville, UT, USA, was utilized for statistical analysis for assessing the data gathered from the study. Quantitative factors were expressed in terms of means, standard deviations, medians, and minimum and maximum values while evaluating the study’s data, and qualitative variables were expressed using descriptive statistical methods, including frequencies and percentages. The Shapiro–Wilk test and boxplot graphs were used to evaluate the conformity of the data to the normal distribution. The Games–Howell test was used to determine which group was responsible for the difference, and the One-Way ANOVA test was used to compare the normally distributed variables among the three groups. For the comparison of non-normally distributed variables, the three groups were compared using the Kruskal–Wallis test, and the Dunn test was used to identify which group was responsible for the difference. To compare the qualitative data, Fisher’s Freeman–Halton test was employed. To assess the relation between Vit-D levels and all-cause mortality, univariate and multivariable Cox proportional hazard regression analyses were performed. The variables in the univariate Cox proportional hazard regression with a *p* value of < 0.25 were entered in the multivariable proportional hazard regression. The association between Vit-D levels and all-cause mortality was quantified based on the hazard ratio. The results were assessed using a significance level of *p* < 0.05 and the 95% confidence interval. Multinomial regression analysis was used to estimate the association between vitamin D levels and ACS. The ratio of the independent variables to explain non-STEMI or UA was calculated as Nagelkerke R^2^ = 0.286.

## 3. Results

There was a total of 408 patients in the study, 49% of whom were male. The average age of the patients was 64 years. The patients underwent PTCA and stenting in 52% of the cases, and 43% of them had previously undergone coronary artery bypass surgery. The patients were divided into groups according to their Vit-D levels: group 1: <10 ng/mL (*n* = 98); group 2: 10–20 ng/mL (*n* = 195); group 3: >20 ng/mL (*n* = 115). Multi-vessel disease was present in 22 patients who underwent stenting. The vitamin D levels of six of them were over 20 ng/mL, eight of them were between 10 and 20 ng/mL, and eight of them were lower than 10 ng/mL. Intervention was performed on the LAD of 129 patients, on the RCA of 65 patients, on the CX of 29 patients, on the diagonal artery of four patients, on the intermediate artery of five patients, and on the saphenous vein graft of three patients. There was no difference between the groups in the variety of vessels treated (*p* > 0.05). The clinical characteristics and test results of the study groups are presented in Table 1, Table 2 and Table 3.

The number of men was significantly higher in group 3 than in groups 1 and 2 (*p* = 0.001). At the same time, the patients in this group were found to be older than those in groups 1 and 2 (*p* = 0018; *p* = 0.001, respectively). There was no difference between the groups. The BMI, hypertension, smoking, follow-up time, and use of acetylsalicylic acid, ACE inhibitors, and ARB data were all studied. The difference was in the drug use; the patients in group 2 used more statins than those in group 3 (*p* = 0.045), while the dose of beta-blockers used by the group 1 patients was higher than those in group 2 and group 3 (*p* = 0.029). The diabetes rate of the cases in group 1 was found to be significantly higher than that of the cases in group 2 (group 1: 59%; group 2: 41%; *p* = 0.01). There was no difference between the groups in terms of stents or CABG intervention. When the stents that were implanted in the patients were examined, there was no difference between the groups in terms of DES. However, the number of bare metal stents in group 3 was found to be significantly lower than those in the other groups (*p* = 0.013). Because the information about the type of stents previously placed in some patients could not be accessed in their files, they were named as undefined.

Although the glucose, HbA1C, HDL, and LDL values of the patients were similar, the triglyceride values were different. This difference was found between group 3 and groups 1 and 2, in which the triglyceride values of group 3 were found to be significantly lower than those of group 1 and group 2 (*p* = 0.038). Again, the calcium values in group 3 were found to be significantly higher than those in groups 1 and 2 (*p* = 0.002).

The lymphocyte, white blood cell, basophil, neutrophil, monocyte, and MPV (mean platelet volume) counts were similar between the groups. Although the neutrophil-to-lymphocyte ratio (NLR), platelet-to-lymphocyte ratio (PLR), MPV-to-lymphocyte ratio, and MPV-to-neutrophil ratio, which are predictors of inflammation, were similar between the groups, the CRP values were different between the groups. The CRP values of the patients in group 3 were found to be significantly lower than those of the group 1 and group 2 patients (*p* = 0.006).

**Figure 2:**
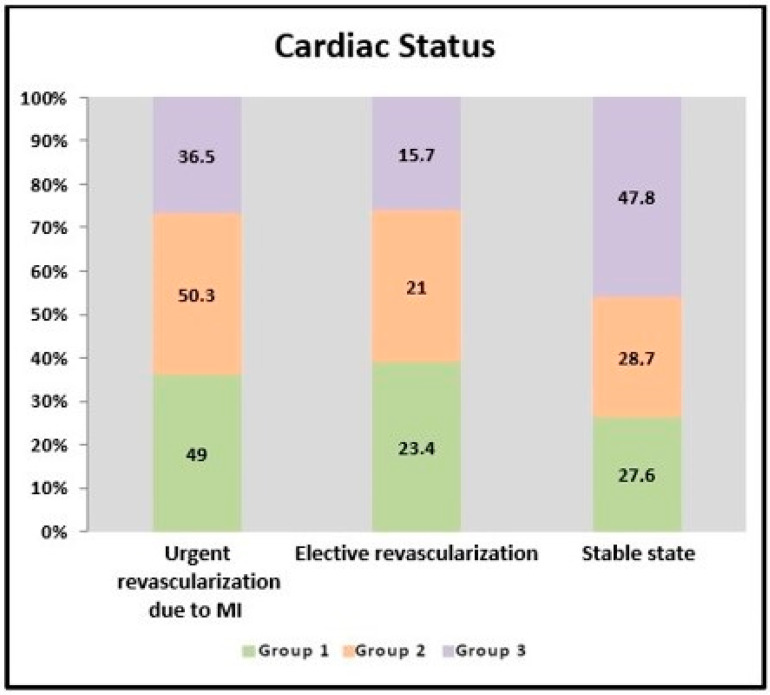
Cardiac statuses of the groups.

When the impact of Vit-D levels on clinical events was evaluated, it was observed that the patients in group 3 remained more stable than the patients in group 1 and group 2 (group 1: 28%; group 2: 29%; group 3: 48%; *p* = 0.006) (Figure 2). Emergency revascularization due to myocardial infarction was performed in at least 36% of the patients in group 3 (group 1: 49%; group 2: 50%; group 3: 36%; *p* = 0.006). It was observed that they experienced fewer non-STEACS and unstable angina events (group 1: 49%; group 2: 38%; group 3: 27%; *p* = 0.001). If the vitamin D level was below 10, the risk of non-STEACS increased by 2.53 times, [ODDS: 2.530 (95% CI: 1.238–5.196)] (*p* = 0.011; *p* < 0.05); and when the vitamin D level was between 10 and 20, the risk of non-STEACS increased by 2083 times [ODDS: 2.083 (95% CI: 1.117–3.884)] (*p* = 0.021; *p* < 0.05) (adjusted for age, gender, CRP levels, creatinine, Vitamin D levels, and diabetes). The rate of STEMI in all three groups was not different. Fewer patients in group 3 required elective coronary revascularization, and this value was not statistically significant. The all-cause mortality in all three groups was similar. In the univariate analysis, variables with a *p*-value of <0.25 were entered in the multivariable Cox regression analysis (Table 4). In the multivariable Cox regression analysis, there was no significant association between all-cause mortality and Vit-D levels (HR = 1.02; 95% CI: 0.98–1.07; *p* = 0.316) after adjusting by age, hypertension, HbA1c, HDL, hemoglobin, creatinine, pro-BNP, and EF (Table 5).

## 4. Discussion

In our study, the effects of Vit-D levels on long-term cardiovascular events in patients with angiographically detected coronary artery disease or who underwent coronary intervention (CABG or underwent PTCA and stenting) were as follows:

1. Vit-D levels were not related to all-cause mortality.
2. A Vit-D level below 20 ng/mL was associated with increased non-STEACS.
3. In patients with coronary artery disease, a Vit-D level above 20 ng/mL was associated with the patients remaining more stable throughout the six-year follow-up.
4. No association was found between STEMI and Vit-D levels.
5. The need for elective revascularization increased as the Vit-D level decreased, but this was not statistically significant.

When we reviewed the literature for coronary events associated with low Vit-D levels, we found the following studies:

Jaiswal et al., in a meta-analysis of eight studies involving 426,039 patients, pointed out that low blood levels of Vit-D are not associated with all-cause mortality. However, low vitamin D levels have been associated with major adverse coronary events (MACEs). Previous PCIs (59% and 28%) were only available in two studies (number of patients was 485 and 101, respectively) that constituted this meta-analysis. In this meta-analysis, of the patient population with a mean age of 65, vitamin D deficiency was not associated with all-cause mortality, but a difference was observed in the sub-group. For all-cause mortality, chronic kidney disease populations with suboptimal Vit-D levels were found to have higher mortality rates than the populations with adequate Vit-D levels (OR 2.73; 95% CI from 1.5 to 4.95) [19].

In our study, although deaths from all causes were less common at six years when the Vit-D levels remained below 20 ng/mL, this created a meaningless trend. The contribution of our study to the literature is that all the patients had angiographically proven coronary artery disease. In the present study, the creatinine value of 27 patients was above normal. Fourteen of these patients died, and their distribution, according to their Vit-D levels, was balanced (accounting for five, four, and five people in groups 1, 2, and 3, respectively). The findings of the current investigation, which were in line with those of Jaiswal et al.’s meta-analysis, showed that low Vit-D levels had no impact on the all-cause mortality of patients with established coronary artery disease. The few patients with high creatinine values in our study may be responsible for the lack of an association between Vit-D levels and mortality in the patients with high creatinine values.

In the VITAL study conducted by Manson et al., daily use of 2000 U of vitamin D had no effect on mortality and cardiovascular events in healthy adults at the end of five years. Patients with chronic renal failure were not included in this study. The mortality results in the VITAL study were consistent with those in our study. Although our patient group in our study is a group of patients with proven coronary artery disease, the patient group of the VITAL study is healthy individuals. Therefore, the difference in our results may be due to our patient population difference. Additionally, the use of single dose of vitamin D in this study may have masked the effects of vitamin D that may occur between individuals [20].

Another meta-analysis of 13 studies examining 17,892 patients with CAD found an adjusted RR of 1.33 (95% CI: 1.18–1.49) for MACEs among patients with low Vit-D levels. In this study, MACEs comprised death, revascularization, stroke, non-fatal myocardial infarction, and heart failure. The patient population of the six studies in this meta-analysis were patients with acute coronary syndrome. In one study, the patient profile was made up of patients with stable CAD. Acute coronary syndrome patients comprised the patient population of the six studies included in this meta-analysis. Patients with stable CAD made up the patient profile in one trial. In this study, the RR was found to be 0.83 (95% CI from 0.37 to 1.86) in MACEs in patients with low Vit-D levels. All-cause mortality was also found to have an RR of 0.94 (95% CI from 0.39 to 2.26) [21]. The patient group in our study was similar to the stable coronary artery patient profile in a study in this meta-analysis. Mortality in our patients was not related to vitamin D levels, but unlike that study, urgent revascularization due to MI was found to be significantly higher in patients with low vitamin D levels.

In follow-up research on 18,000 healthy male participants, those with 25-hydroxyl Vit-D levels below 15 ng/mL had a relative risk of myocardial infarction of 2.42 (95% CI from 1.53 to 3.84), which was compared to those with levels over 30 ng/mL after 10 years [22]. In our study, non-STEACS was more common when vitamin D levels were below 20 ng/mL.

In different studies, the vitamin D deficiency level of hospitalized patients with a myocardial infarction was in the range 70–95%. In addition, the frequency of severe deficiencies was 10–40% [23]. In our study, patients with severely low vitamin D levels (<10 ng/mL) accounted for 26% of the patients with acute myocardial infarctions. In a study by Verdoia et al. on 705 patients undergoing angiography, after three years of follow-up, low Vit-D levels were associated with poor survival and high rates of recurrent cardiovascular events [24]. Our study was methodologically different from this study. A total of 60% of the patients underwent coronary angiography owing to ACS. Our patient population was from the outpatient clinic. In this study, the rate of patients with coronary bypass surgery was 11%, and the rate of patients with previous stenting was 35%. In our study, 44% of the patients had coronary bypass surgery, 52% had previous stenting, and the interventional rate was higher in our study.

In a study conducted by Beska et al. on 298 elderly (mean age 80.5 ± 4.8 years) patients with non-STEACS, a relationship was found between low-dose Vit-D supplementation and prior MIs, but no relationship was found between low-dose Vit-D supplementation and adverse events at the end of the first year. In that study, 11.6% of the patients took Vit-D at the beginning of the study, and this value increased to 19.4% at the end of the study [25]. In our study, the number of patients over the age of 80 was only 29 (distributed into groups as follows: 6, 11, and 12 in groups 1, 2, and 3, respectively), and five patients died (distributed into groups as follows: 1, 1, and 3 in groups 1, 2, and 3, respectively). Myocardial infarction was observed in 12 patients (distributed into groups as follows: 4, 8, and 4 in groups 1, 2, and 3, respectively). In our study, fewer myocardial infarctions were observed in elderly patients when vitamin D levels were above 20 ng/mL. However, owing to the small number of patients, statistical evaluation was not possible.

Two epidemiological studies investigated the relationship between vitamin D levels and cardiovascular events and mortality. In one of these studies, Cannistraci et al. discovered that during the summer, a portion of the STEMIs drastically changed from the 6:00–18:00 (diurnal) interval and that the difference between the numbers of STEMIs in the 6:00–18:00 (diurnal) interval and the 18:00–6:00 (nocturnal) interval was diminished. The term “summer shift” is used to describe this phenomenon. Owing to its high reliance on the quantity and quality of sunshine exposure, Vit-D is sometimes known as the “sun vitamin”. According to this epidemiological investigation, Vit-D levels and the “summer shift” are correlated [26]. In our study, STEMI was not found to be associated with vitamin D levels. Procedure-related events may have contributed to the results, as 52% of our patients had previously received stents.

In another epidemiological study, excessive winter mortality (EWM) due to acute MIs has been observed in England and Wales. In all the periods, patients aged 75 years and older had the highest absolute EWM, and the lowest mortality was recorded in August. The researchers hypothesized that 25-hydroxyl Vit-D concentrations above 36 ng/mL may reduce the winter mortality rate [27]. Our study was methodologically different from this study. Vitamin D levels were classified as <10, 10–20, and >20 ng/mL, and there were only 14 patients with vitamin D levels over 36 in our study.

In a study by Afifeh et al., lower calcitriol levels were linked to a non-significant trend for STEMI presentation in 228 ACS patients receiving early invasive treatment [28]. Although there was no association between STEMI and Vit-D levels in the present study, the incidences of USAP and non-STEMI declined significantly when the Vit-D levels exceeded 20 ng/mL. The rate of not having a myocardial infarction was significantly higher in group 3, but this difference is due to the reduced probability of having a USAP and non-STEMI. The lack of association between Vit-D levels and STEMI in our study may be due to the increased possibility of stent thrombosis because 52% of our patient group had previously undergone PTCA and stenting. It has been reported in previous studies that stent thrombosis can also be caused by interventional procedures and generally progresses to STEMI [29].

In the current study, there was a statistically insignificant increase in the rate of elective revascularization at Vit-D levels below 20 ng/mL. In another study, when the Vit-D levels were below 11.3 ng/mL, the incidence of coronary stent restenosis increased 3.4-fold (area under the curve = 0.651; 95% CI from 0.529 to 0.773; *p* = 0.02) [30]. In another study, VDR gene polymorphisms were associated with in-stent restenosis following percutaneous coronary intervention [23]. A statistical significance may not have been observed in our study because the number of elective revascularizations was low.

Other studies have shown a potential link between Vit-D levels and stable coronary artery disease. The possible mechanisms by which Vit-D deficiency is related to deterioration in stable CAD are as follows: It may be directly related to the activation of renin–angiotensin and an increase in inflammation or indirectly related to the increase in parathormone caused by the decrease in calcium [31]. PTH can stimulate cytokine release from lymphocytes and, thus, may affect the cardiovascular system via pro-inflammatory effects. In a meta-analysis of 12 cohort studies, it was found that an increase in the PTH level increased the number of total cardiovascular events by 1.45 times [32]. Furthermore, higher PTH concentrations might increase arterial stiffness [33]. In addition, PTH has been shown to predict primary ischemic events when the calcidiol level falls below 20 ng/mL [34].

In our study, when the Vit-D level fell below 20 ng/mL, the blood calcium level decreased significantly. This significant decrease in mineral metabolism may have caused an increase in the PTH levels. At the same time, the severely low inflammatory status among the patients in group 3 compared to those in the other groups may have helped to maintain their clinically stable status.

Additionally, in our study, most patients with insufficient vitamin D levels were female. As the severity of the deficiency increases, the proportion of female patients also increases. According to the literature, being female is an additional factor for vitamin D deficiency, which explains the excess number of females with low vitamin D levels in our study [8]. Likewise, in our study, the diabetic patient population was found to be higher in the group with severe vitamin D deficiency. This may be because vitamin D deficiency impairs insulin synthesis and insulin sensitivity, thus creating a predisposition to develop diabetes [31].

Compared with the literature in general, our study is substantially different in terms of the high number of patients with an interventional history in the patient population. In addition, the selection of patients who experienced a single type of event was also important for preventing the effect of different types of consecutive events on the results.

However, our study has some limitations. This study is an observational cross-sectional, retrospective, single-center study, and there may be a selection bias based on the patients’ characteristics. Although extrinsic factors affected the endpoints, we tried to apply strict exclusion criteria to obtain a homogeneous population. In our study, the numbers of women and diabetic patients were higher in the patient group with low vitamin D levels. This may have contributed to the higher incidence of myocardial infarctions in this group. Again, because the stent types of some of the patients’ previous stent procedures were not available, this represents a limitation on the results. Because it was a retrospective study, costly tests, such as lipoprotein A and homocysteine levels, were not used in routine practice, and the effect of these factors on our results could not be evaluated. Some other limitations include the fact that PTH levels were not examined in the study and neither was the effect of seasonal changes on Vit-D levels. In addition, the effects of the risks of drug discontinuation for various reasons on cardiovascular events during patient follow-ups were another limitation of our study.

## 5. Conclusions

Our study did not find a relationship between vitamin D levels and mortality in patients who had previously undergone coronary bypass, PTCA, or had documented coronary artery disease. However, increased incidences of non-STEMI and UA were observed in these patients when vitamin D levels were below 20 ng/mL. It is also interesting that there was no relationship between STEMI and vitamin D levels in our study. The reason for this may have been the contribution of possible procedural events due to the higher number of previous stent and previous CABG patients in our study compared to other studies. The increased likelihood of non-STEMI and UA in patients with vitamin D deficiency may provide an explanation for the increase in the urgent need for revascularization due to myocardial infarction in these patients. Another important result of our study was that patients without a vitamin D deficiency remained stable for a long period of up to six years.

Additionally, this study observed a slight increase in elective revascularization in patients with low vitamin D levels, which was not statistically significant; the low number of elective revascularizations in our study may have affected this increase to reach significant levels.

Our study needs to be supported by prospective, comprehensive studies that include more chronic kidney disease patients; measuring the PTH, lipoprotein A, and homocysteine levels; and standardizing seasonal changes.

## Figures and Tables

**Table 1:** Comparison of clinical and demographic characteristics of groups separated according to vitamin D levels.

		Vitamin D Levels			*p*
		Group 1	Group 2	Group 3	
		(<10 ng/mL)	(10–20 ng/mL)	(>20 ng/mL) (*n* = 115)	
		(*n* = 98)	(*n* = 195)		
Sex (%)	Female	64.3	55.4	33.9	^a^ 0.001 **
	Male	35.7	44.6	66.1	
Age	mean ± SD	62.63 ± 11.30	61.96 ± 10.42	66.77 ± 10.55	^b^ 0.001 **
BMI (kg/m^2^)	mean ± SD	28.90 ± 5.90	27.90 ± 4.50	28.20 ± 4.50	^b^ 0.481
Hypertension (%)		55.1	59	58.3	^a^ 0.810
Diabetes (%)		59	40.5	47	^a^ 0.011 *
Smokers (%)		11.2	18.5	10.4	^a^ 0.097
Acetylsalicylic acid (%)		84.7	87.7	84.3	^a^ 0.627
Beta blocker (%)		95.9	88.7	85.2	^a^ 0.029 *
ACEI, ARB (%)		77.6	80.5	68.7	^a^ 0.062
Statin (%)		77.6	78.5	66.1	^a^ 0.045 *
PPI (%)		88.8	86.7	75.7	^a^ 0.019 *
Mediterranean type diet (%)		87	86	84	^a^ 0.828
Sedentary lifestyle (%)		10	9	13	^a^ 0.568

^a^ Fisher’s Freeman–Halton Test. ^b^ One-Way ANOVA Test and Bonferroni Test. * *p* < 0.05; ** *p* < 0.01. BMI: Body Mass Index. ACEI: Angiotensin-Converting Enzyme Inhibitor. ARB: Angiotensin Receptor Blocker. PPI: Proton-Pump Inhibitor.

**Table 2:** Comparison of cardiac statuses and treatments according to groups.

		Group 1	Group 2	Group 3	*p*
		(*n* = 98)	(*n* = 195)	(*n* = 115)	
Cardiac Status (%)	Urgent revascularization due to MI	49	50.3	36.5	^a^ 0.006 **
	Elective revascularization	23.4	21	15.7	^a^ 0.331
	Stable state	27.6	28.7	47.8	^a^ 0.006 **
ACS (%)	STEMI	23.4	34.4	26	^a^ 0.101
	Non-STEACS, UA	49	37.9	27	^a^ 0.001 **
Previous Stent (%)		58.2	55.4	43.5	^a^ 0.059
Previous Stent Type (%)	BMS	22.4	28.1	12.4	^a^ 0.013 *
	DES	25.5	22.4	21.2	^a^ 0.719
	Undefined	10.3	4.9	9.9	^a^ 0.126
Previous CABG (%)		42.9	37.4	48.7	^a^ 0.152
Mortality (%)		24.5	13.8	17.4	^a^ 0.082
Follow-up (months)	mean ± SD	57.04 ± 18.48	58,27 ± 18.12	53.73 ± 19.10	^c^ 0.059

^a^ Fisher’s Freeman–Halton Test. ^c^ Kruskal–Wallis Test. * *p* < 0.05; ** *p* < 0.01. CABG: coronary artery bypass grafting. MI: myocardial infarction. BMS: bare metal stent. DES: Drug-Eluting Stent. ACS: acute coronary syndrome. STEMI: ST-elevated myocardial infarction. Non-STEACS: non-ST-elevated acute coronary syndrome. UA: unstable angina.

**Table 3:** Comparison of laboratory measurements in groups separated according to vitamin D levels.

		Total	Vitamin D Levels			*p*
			Group 1	Group 2	Group 3	
			<10 ng/mL	10–20 ng/mL	>20 ng/mL	
			(*n* = 98)	(*n* = 195)	(*n* = 115)	
Fasting glucose (mg/dL)	mean ± SD	124.90 ± 53.80	132.70 ± 58.60	123.40 ± 47.60	120.90 ± 59.20	^c^ 0.135
HbA1c (%)	mean ± SD	6.60 ± 1.60	7.03 ± 2.03	6.40 ± 1.60	6.40 ± 1.20	^c^ 0.186
Triglycerides (mg/dL)	mean ± SD	166.1 ± 111.6	176.4 ± 118.2	173.2 ± 122.3	145.6 ± 80.9	^c^ 0.038 *
HDL (mg/dL)	mean ± SD	41.50 ± 12.10	40.70 ± 10.80	41.10 ± 11.60	42.80 ± 13.60	^b^ 0.364
LDL (mg/dL)	mean ± SD	120.40 ± 53.30	120.40 ± 38.70	119.60 ± 65.40	121.80 ± 39.40	^b^ 0.941
WBC (10^3^/mm^3^)	mean ± SD	8.40 ± 2.30	8.60 ± 2.60	8.40 ± 2.50	8.20 ± 1.90	^b^ 0.464
Hemoglobin (g/dL)	mean ± SD	13.30 ± 1.90	12.80 ± 2	13.50 ± 1.80	13.40 ± 1.80	^b^ 0.012 *
Platelet (10^3^/mm^3^)	mean ± SD	243 ± 73.40	247.70 ± 76.40	233.80 ± 72	254.60 ± 71.50	^b^ 0.041 *
Neutrophil (10^3^/mm^3^)	mean ± SD	5.50 ± 2.60	5.70 ± 2.40	5.50 ± 3	5.20 ± 1.80	^c^ 0.763
Lymphocyte (10^3^/mm^3^)	mean ± SD	2.20 ± 0.80	2.20 ± 0.90	2.10 ± 0.80	2.10 ± 0.60	^b^ 0.719
MPV (fL)	mean ± SD	9 ± 1.40	8.90 ± 1.40	8.90 ± 1.30	9.10 ± 1.40	^b^ 0.468
Platelet/Lymphocyte	mean ± SD	127.80 ± 78.10	128.50 ± 61.40	128.20 ± 96.90	126.50 ± 50.70	^c^ 0.694
NLR	mean ± SD	3.10 ± 3.70	3.10 ± 2.10	3.50 ± 5.10	2.60 ± 1.40	^b^ 0.186
MPV/Lymphocyte	mean ± SD	4.90 ± 2.80	4.70 ± 2.10	5.20 ± 3.50	4.60 ± 1.60	^b^ 0.173
MPV/Neutrophil	mean ± SD	1.90 ± 0.80	1.80 ± 0.70	1.90 ± 0.90	1.90 ± 0.70	^b^ 0.421
Monocyte (10^3^/mm^3^)	mean ± SD	0.60 ± 0.20	0.60 ± 0.20	0.60 ± 0.20	0.60 ± 0.20	^b^ 0.463
Eosinophil (10^3^/mm^3^)	mean ± SD	0.20 ± 0.10	0.10 ± 0.10	0.20 ± 0.10	0.20 ± 0.10	^b^ 0.049 *
Basophil 10^3^/mm^3^	mean ± SD	0.01 ± 0.10	0.10 ± 0.10	0.10 ± 0.10	0.10 ± 0.10	^c^ 0.892
Calcium (mg/dL)	mean ± SD	9.20 ± 0.70	9.10 ± 1.10	9.20 ± 0.60	9.40 ± 0.50	^b^ 0.002 **
Phosphor (mg/dL)	mean ± SD	3.70 ± 3	3.60 ± 0.90	3.80 ± 4.30	3.60 ± 0.80	^b^ 0.865
Uric acid (mg/dL)	mean ± SD	5 ± 2.40	4.90 ± 2.50	4.90 ± 2.40	5.30 ± 2.40	^b^ 0.262
CRP (mg/L)	mean ± SD	1.10 ± 3.10	0.90 ± 1.40	1.40 ± 4.20	0.70 ± 1.20	^c^ 0.006 **
Creatinine (mg/dL)	mean ± SD	1 ± 0.50	1 ± 0.60	1 ± 0.50	1.10 ± 0.50	^c^ 0.017 *
BNP (pg/mL)	mean ± SD	353.2 ± 962.7	743.2 ± 1826	213.5 ± 287.7	245.7 ± 317	^c^ 0.089
EF (%)	mean ± SD	50.70 ± 10.50	50.70 ± 11.20	50.70 ± 10.30	50.70 ± 10.40	^b^ 0.999

^b^ One-Way ANOVA Test and Games–Howell Test. ^c^ Kruskal–Wallis Test and Dunn–Bonferroni Test. * *p* < 0.05; ** *p* < 0.01. WBC: white blood cell. LDL: Low-Density Lipoprotein Cholesterol. HDL: High-Density Lipoprotein Cholesterol. NLR: neutrophil-to-lymphocyte ratio. CRP: C-reactive protein. MPV: mean platelet volume. EF: Ejection Fraction. HbA1c: Glycated Hemoglobin. BNP: Brain Natriuretic Peptide.

**Table 4:** Baseline characteristics according to status (dead/alive).

Variables	Alive (*n* = 330)	Dead (*n* = 78)	*p*
Age, years	63 ± 11	67 ± 12	<0.001
Sex, Male, *n* (%)	158 (48%)	40 (51%)	0.600
Vitamin D level (ng/mL)	17 ± 11	15 ± 9	0.212
Vitamin D category, *n* (%)			0.077
<10 ng/mL	72 (22%)	26 (33%)	
10–20 ng/mL	165 (50%)	30 (39%)	
>20 ng/mL	93 (28%)	22 (28%)	
Smoking, *n* (%)	50 (15%)	14 (18%)	0.537
Hypertension, *n* (%)	178 (54%)	58 (74%)	<0.001
BMI (kg/m^2^)	28.0 ± 4.6	28.9 ± 5.3	0.546
Diabetes, *n* (%)	142 (43%)	44 (56%)	0.037
Fasting glucose (mg/dL)	122 ± 49	139 ± 72	0.039
HbA1c (%)	6.40 ± 1.50	7.22 ± 1.92	<0.001
Triglycerides (mg/dL)	166 ± 115	171 ± 86	0.174
HDL (mg/dL)	42 ± 12	39 ± 11	0.014
LDL (mg/dL)	119 ± 41	123 ± 89	0.463
WBC (10^3^/mm^3^)	8.33 ± 2.29	8.71 ± 2.70	0.179
Hemoglobin (g/dL)	13.49 ± 1.79	12.68 ± 2.19	0.003
Platelet (10^3^/mm^3^)	241 ± 71	252 ± 83	0.366
NLR	3.04 ± 3.92	3.45 ± 2.38	0.005
CRP (mg/L)	1.07 ± 3.18	1.13 ± 2.07	0.115
Creatinine (mg/dL)	0.99 ± 0.48	1.21 ± 0.65	<0.001
BNP (pg/mL)	302 ± 1037	533 ± 623	0.003
LVEF (%)	52 ± 9	46 ± 14	<0.001
Previous Stent, *n* (%)	172 (52%)	47 (60%)	0.186
Previous CABG, *n* (%)	142 (43%)	24 (31%)	0.049
Acetylsalicylic acid, *n* (%)	283 (86%)	71 (91%)	0.212
Beta blocker, *n* (%)	294 (89%)	73 (94%)	0.237
ACEI, ARB, *n* (%)	251 (76%)	65 (83%)	0.162
Statin, *n* (%)	251 (76%)	59 (76%)	0.989
PPI, *n* (%)	274 (83%)	70 (90%)	0.163

BMI: Body Mass Index; HbA1c: Glycated Hemoglobin; HDL: High-Density Lipoprotein Cholesterol; LDL: Low-Density Lipoprotein Cholesterol; WBC: white blood cell; NLR: neutrophil-to-lymphocyte ratio; CRP: C-reactive protein; BNP: Brain Natriuretic Peptide; LVEF: Left Ventricular Ejection Fraction; CABG: Coronary Artery Bypass Grafting; ACEI: Angiotensin-Converting Enzyme Inhibitor; ARB: Angiotensin Receptor Blockers; PPI: Proton Pump Inhibitor.

**Table 5:** Predictors of long-term death by multivariable Cox regression analysis.

	Hazard Ratio	95% CI of Hazard Ratio	*p*
Age	1.02	1.00, 1.05	0.104
Vitamin D level	1.00	0.97, 1.03	0.993
Diabetes	1.41	0.83, 2.41	0.207
Hypertension	1.51	0.83, 2.74	0.176
HDL cholesterol	0.99	0.96, 1.01	0.282
Hemoglobin	0.88	0.77, 1.01	0.078
Creatinine	1.27	0.90, 1.79	0.181
LVEF	0.97	0.95, 0.99	0.007
Triglyceride	1.00	1.00, 1.00	0.991
WBC	1.03	0.92, 1.15	0.596
CRP	1.02	0.95, 1.11	0.53
PPI	1.02	0.45, 2.35	0.954

HDL: High-Density Lipoprotein Cholesterol; WBC: White Blood Cell; CRP: C-Reactive Protein; LVEF: Left Ventricular Ejection Fraction; PPI: Proton Pump Inhibitor.

## Data Availability

Available upon request.

## References

[1] Husain K., Hernandez W., Ansari R., Ferder L. (2015). Inflammation, oxidative stress and renin angiotensin system in atherosclerosis. World J. Biol. Chem..

[2] Cimmino G., Morello A., Conte S., Pellegrino G., Marra L., Golino P., Cirillo P. (2020). Vitamin D inhibits Tissue Factor and CAMs expression in oxidized low-density lipoproteins-treated human endothelial cells by modulating NF-KB pathway. Eur. J. Pharmacol..

[3] Yin K., Agrawal D. (2014). Vitamin D and inflammatory diseases. J. Inflamm. Res..

[4] Siadat D.Z., Kiani K., Sadeghi M., Shariat A., Farajzedegan Z., Kheirmand M. (2012). Association of Vitamin D deficiency and coronary artery disease with cardiovascular risk factors. J. Res. Med. Sci..

[5] Surdu A.M., Pinzariu O., Ciobanu D.M., Negru A.G., Cainap S.S., Lazea C., Iacob D., Saraci G., Trinescu D., Borda H.M. (2021). Vitamin D and its role in the lipid metabolism and the development of atherosclerosis. Biomedicines.

[6] Cimmino G., Conte S., Morello M., Pellegrino G., Marra L., Morello A., Nicoletti G., De Rosa G., Golino P., Cirillo P. (2022). Vitamin D Inhibits IL-6 Pro-Atherothrombotic Effects in Human Endothelial Cells: A Potential Mechanism for Protection against COVID-19 Infection?. J. Cardiovasc. Dev. Dis..

[7] Mozos I., Marginean O. (2015). Links between Vitamin D Deficiency and Cardiovascular Disease. Biomed. Res. Int..

[8] Mandarino R.N., Junior F.C., Salgado J.L., Lages J.S., Filho N.S. (2015). Is Vitamin D deficiency a new risk factor for cardiovascular disease?. Open Cardiovasc. Med. J..

[9] Gardner G., Chen S., Glenn D.J. (2013). Vitamin D and the heart. Am. J. Physiol. Regul. Comp. Physiol..

[10] Milazzo V., Metrio M., Cosentino N., Marenzi G., Tremoli E. (2017). Vitamin D and acute myocardial infarction. World J. Cardiol..

[11] Ho J.S., Cannaday J.J., Barlow C.E., Reinhardt D.B., Wade W.A., Ellis J.R. (2015). Low 25-0H Vitamin D levels are not associated with coronary artery calcium or obstructive stenosis. Coron. Artery Dis..

[12] Centers for Disease Control and Prevention (CDC) Excessive Alcohol Use. www.cdc.gov/chronicdisease/resources/publications/factsheets/alcohol.htm.

[13] Collet J.P., Thiele H., Barbato E., Barthe O., Bauersachs J., Bhatt D.L., Dendale P., Dorobantu M., Edvardsen T., Folliguet T. (2021). 2020 ESC Guidelines for the management of acute coronary syndromes in patients presenting without persistent ST-segment elevation. Eur. Heart J..

[14] Elsayed N.A., Aleppa G., Arada V.R., Bannuru R.R., Brown F.M., Bruemmer D., Collins B.S., Hillard M.E., Isaacs D., Johnson E.L. (2023). Glycemic targets: Standards of care in Diabetes-2023. Diabetes Care.

[15] Lewington S., Clarke R., Qizilbash N., Peto R., Collins R. (2002). Prospective Studies Collaboration. Age-specific relevance of usual blood pressure to vascular mortality: A meta-analysis of individual data for one million adults in 61 prospective studies. Lancet.

[16] Eklund C., Elfström M., Wagert P.H., Söderlund A., Gustavsson A., Gustavsson C., Cederbom S., Thunborg C., Lööf H. (2021). The meaning of sedentary behavior as experienced by people in the transition from working life to retirement: An empirical phenomenological study. Phys. Ther..

[17] Mancia G., Kreutz R., Brunströmc M., Burnierd M., Grassie G., Januszewiczf A., Muiesang A.L., Tsioufish K., Agabiti-Roseii E., Algharablyb E.A. (2023). 2023 ESH Guidelines for the management of arterial hypertension. J. Hypertens..

[18] Grundy S.M., Stone N.J., Bailey A.L., Beam C., Birtcher K.K., Blumenthal B.S., Braun L.T., Ferranti S., Tommasino J.F., Forman D.E. (2019). 2018 AHA/ACC/AACVPR/AAPA/ ABC/ACPM/ADA/AGS/APhA/ASPC/ NLA/PCNA Guideline on the Management of Blood Cholesterol. J. Am. Coll. Cardiol..

[19] Jaiswal V., Ishak A., Ang S.P., Pokhrel N.B., Shama N., Lnu K., Varghese J.S., Storozhenko T., Chia J.E., Naz S. (2022). Hypovitaminosis D and cardiovascular outcomes: A systematic review and meta-analysis. Int. J. Cardiol. Heart Vasc..

[20] Manson J.E., Cook N.R., Lee I.M., Christen W., Bassuk S.S., Mora S., Gibson H., Gordon D., Copeland T., VITAL Research Group (2019). Vitamin D Supplements and Prevention of Cancer and Cardiovascular Disease. N. Engl. J. Med..

[21] Zhang H., Wang P., Sun Y., Wang X., Fan Y. (2022). Predictive value of 25-hydroxy Vitamin D level in patients with coronary artery disease: A meta- analysis. Front. Nutr..

[22] Nudy M., Krakowski G., Ghahramani M., Ruzieh M., Joy A. (2020). Vitamin D supplementation, cardiac events and stroke: A systematic review and meta-regression analysis. Int. J. Cardiol. Heart Vasc..

[23] Cosentino N., Campadonice J., Milazzo V., Metrio Mi Brambilla M., Camera M., Marenzi G. (2021). Vitamin D and cardiovascular disease: Current evidence and future perspectives. Nutrients.

[24] Verdoia M., Nardin M., Rolla R., Negro F., Gioscia R., Afifeh A.M.S., Viglione F., Suryapranata H., Marcolongo M., Luca G. (2021). Prognostic impact of Vitamin D deficiency in patients with coronary artery disease undergoing percutaneous coronary intervention. Eur. J. Intern. Med..

[25] Beska B., Chan D., Gu S., Qiu W., Mossop H., Neely D., Kunadian V. (2018). The association between Vitamin D status and clinical events in high-risk older patients with non- ST elevation acute coronary syndrome undergoing invasive mangement. PLoS ONE.

[26] Cannistraci C.V., Nieminen T., Nishi M., Khachigian L.M., Viikila J., Laina M., Cianflone D., Maseri A., Yeo K.K., Bhindi R. (2018). Summer Shift: Apotential sunshine on the time onset of ST-elevation acute myocardial infarction. J. Am. Heart Assoc..

[27] Ogbegor O., Odugbemi B., Maheswaran R., Kavya P. (2018). Seasonal variation in mortality secondary to acute myocardial infarction in England and Wales: A secondary data analysis. BMJ Open.

[28] Afifeh A.M., Verdoia M., Nardin M., Negro F., Viglione F., Rolla R., Luca G. (2021). Determinants of vitamin D activation in patients with acute coronary syndromes and its correlation with inflammatory markers. Nutr. Metab. Cardiovasc. Dis..

[29] Byrne R., Joner M., Kastrati A. (2015). Stent thrombosis and restenosis: What have we learned and where are we going? The Andreas Gruntzig Lecture ESC 2014. Eur. Heart J..

[30] Yaman A.E., Ceylan U.S. (2021). The effect of Vitamin D deficiency on the risk and time of stent restenosis after percutaneous coronary angioplasty: Case-control study. Turk. Klin. J. Cardiovasc. Sci..

[31] Pravecek M.K., Arar Z.V., Miskic B., Hadzibegovic I. (2017). Vitamin D deficiency in acute coronary syndrome—Clinically relevant or incidental finding?. Cent. Eur. J. Public. Health.

[32] Folsom A.R., Alanso A., Misialek J.R., Michos E.D., Selvin E., Eckfeldt J.H., Coresh J., Pankow J.S., Lutsey P.L. (2014). Parathyroid hormone concentration and risk of cardiovascular diseases: The atherosclerosis risk in communities (ARIC) study. Am. Heart J..

[33] Gepner A.D., Colangelo L.A., Blondon M., Korcarz C.E., Boer L.H., Kestenbaum B., Siscovick D.S., Kaufman J.D., Liu K., Stein J.H. (2014). 25-hydroxy Vitamin D and parathyroid hormone levels do not predict changes in carotid arterial stiffness: The multi-ethnic study of atherosclerosis. Arterioscler. Thromb. Vasc. Biol..

[34] Landaluce C.G., Acena A., Pello A., Milla J.M., Lorenzo O.G., Tarin N., Cristobal C., Blanco-Colio L.M., Martin-Ventura J.L., Huelmas A. (2021). Parathormone levels add prognostic ability to N-terminal pro-brain natriuretic peptide in stable coronary patients. ESC Heart Fail..

